# General Practitioners’ Knowledge and Concern about Electromagnetic Fields

**DOI:** 10.3390/ijerph111212969

**Published:** 2014-12-12

**Authors:** Gabriele Berg-Beckhoff, Jürgen Breckenkamp, Pia Veldt Larsen, Bernd Kowall

**Affiliations:** 1 Unit of Health Promotion Research, University of Southern Denmark, Esbjerg 6700, Denmark; 2 Department of Epidemiology and International Public Health, University of Bielefeld, Bielefeld 33615, Germany; E-Mail: juergen.breckenkamp@uni-bielefeld.de; 3 Center for Clinical Epidemiology, Odense University Hospital, University of Southern Denmark, Odense 5000, Denmark; E-Mail: pia.veldt.larsen@rsyd.dk; 4 Institute for Medical Informatics, Biometry and Epidemiology, Center of Clinical Epidemiology, University of Duisburg-Essen, Essen 45147, Germany; E-Mail: Bernd.Kowall@uk-essen.de

**Keywords:** general practitioners, electromagnetic fields, risk perception, concern, cross sectional study

## Abstract

Our aim is to explore general practitioners’ (GPs’) knowledge about EMF, and to assess whether different knowledge structures are related to the GPs’ concern about EMF. Random samples were drawn from lists of GPs in Germany in 2008. Knowledge about EMF was assessed by seven items. A latent class analysis was conducted to identify latent structures in GPs’ knowledge. Further, the GPs’ concern about EMF health risk was measured using a score comprising six items. The association between GPs’ concern about EMF and their knowledge was analysed using multiple linear regression. In total 435 (response rate 23.3%) GPs participated in the study. Four groups were identified by the latent class analysis: 43.1% of the GPs gave mainly correct answers; 23.7% of the GPs answered low frequency EMF questions correctly; 19.2% answered only the questions relating EMF with health risks, and 14.0% answered mostly “don’t know”. There was no association between GPs’ latent knowledge classes or between the number of correct answers given by the GPs and their EMF concern, whereas the number of incorrect answers was associated with EMF concern. Greater EMF concern in subjects with more incorrect answers suggests paying particular attention to misconceptions regarding EMF in risk communication.

## 1. Introduction

The level of concern about electromagnetic fields (EMF) is high in the general population. According to a Eurobarometer survey, one third of EU-citizens believe that EMF mobile phone base stations and high tension power lines affect their health “to a large extent” [1], while a further 25% to 37% believe their health might be influenced at least “to some extent”. There are huge between-country differences in the perceived impact rates, ranging from over 80% being concerned (Greece, Italy) to 16% and 17% of concerned citizens in Denmark and Sweden [1].

An important phenomenon related to the concern about EMF health risks is “electromagnetic hypersensitivity” [2], where people relate unspecific symptoms, such as fatigue, dizziness and nausea as well as dermatological symptoms like redness or rashes to EMF-exposure. Some people who perceive themselves as electromagnetically hypersensitive turn to the medical system and consult a GP. A German survey showed that during the last 12 months about 43% of GPs had had at least one patient contact during which electromagnetic fields were named as a potential risk factor [3]. The role of GPs in communicating about EMF health risk is therefore important. A challenge for the GP in this respect is that experimental evidence indicates that people who perceive themselves as electromagnetically hypersensitive are not able to identify presence or absence of EMF under double blind conditions which has led researchers to suggest that adverse symptoms may be due to beliefs in—or expectations of—harm rather than to actual exposure [4,5,6,7]. In epidemiological studies it is also shown that adverse symptoms are only associated with concern about EMF but not with real EMF exposure [8,9]. However, risk factors and causal pathways for the increase in adverse symptoms among patients concerned about EMF are not yet clarified [10].

The societal relevance of GPs’ EMF concerns and knowledge derives from their gate keeper function; they are the first medical professional confronted with patients’ concerns and symptoms regarding EMF. When a GP communicates with a patient on risks in connection with EMF, this message needs to be handled in a constructive manner; however, the communication depends on the GP’s own knowledge and risk perceptions about EMF. The purpose of the present article is to explore GPs’ knowledge about EMF, and to assess whether different knowledge structures or correct/incorrect knowledge are related to the GPs’ concern about electromagnetic fields.

## 2. Method

Samples were drawn from lists of GPs published online by each of the 17 Regional Associations of Statutory Health Insurance Physicians from the 16 federal states in Germany. Almost all German GP practices are covered by these lists. From each of the 17 lists, seven percent samples were drawn at random. The selected GP practices were then randomly assigned to two groups: two thirds (*n* = 1867) received a long self-administered postal questionnaire, one third (*n* = 928) received a short questionnaire. The survey took place between March and May 2008. The long questionnaire covered four pages, the short questionnaire only one page. GPs who had received the long version of the questionnaire and who had not responded after four weeks were sent a reminder and a further questionnaire. In the present analysis only data from the long questionnaire were used, as it comprised detailed questions on knowledge and concern about EMF. Further methods are described in more detail elsewhere [3,11,12].

Knowledge about electromagnetic fields was assessed by seven items, each of which could be answered by either “yes”, “no” or “don’t know”. The seven statements were developed based on an information brochure for GPs about mobile phones and health [13] and were checked for plausibility/factual correctness by physicists of the German Federal Office for Radiation Protection. An English translation of the questions is shown in Table 1. The original German version of the questionnaire is published elsewhere [14]. The answers were categorized as correct, incorrect and “don’t know“. The numbers of correct answers, incorrect answers, and “don’t know”, respectively, were calculated for each participant. Before being given the questions on specific knowledge, participants were asked to rate their own knowledge on the association between health and EMF on a six point scale from “very good” to “very poor”. Further, trust in the authorities providing information on EMF risk was assessed. It was decided to use the World Health Organization (WHO) as the relevant authority because of its international EMF project [15]. WHO considers and evaluates scientific evidence on EMF risk to facilitate dialog between different stakeholders, to help countries to set up EMF legislation, and to provide information on EMF risks communication [16]. Trust in WHO information was measured with a single item on a six-point scale ranging from “very high” to “very low”.

Concern about each of 13 different health risks were to be rated on a four-point scale. The health risks included air pollution, traffic noise, road accidents, drug side effects, smoking, alcohol consumption, consumption of meat from unknown origin, and six items regarding EMF. The six EMF related items concerned radio and television broadcast, home electrical equipment, mobile phone base stations, mobile phones, cordless telephones (DECT standard), and high voltage power lines, respectively. The six EMF items were added up as a score between 6 (not concerned) and 24 points (highly concerned). Only seven persons had not answered one or more of these items. Due to the small number of resulting missing values no imputation was made, instead the seven persons were excluded from the analyses. A correlation matrix was constructed using the Spearman rank correlation coefficients of EMF concern (score from 6 to 24) and the five explanatory variables: number of correct answers (0–7), number of incorrect answers (0–7), number of “don’t know” (0–7), trust in WHO information (score from 0 to 6), and self-estimated knowledge (score from 1 to 6). Cronbach’s alpha was calculated for the six EMF concern items.

**Table 1:** Answers to questions about EMF-knowledge by GPs in Germany in 2008.

Questions	Type of Question *	Correct Answer	Correct Statement		Incorrect Statement		Don’t Know Statement	
			*n*	%	*n*	%	*n*	%
Frequency of 100 Hertz belongs to low-frequency EMF	LF-EMF	Yes	243	58.6	35	8.4	137	33.0
The power of mobile phones is higher the better the EMF reception is.	HF-EMF	No	223	52.7	72	17.0	128	30.3
For the German population the mean EMF exposure is far below the legal limits	EMF Health	Yes	205	48.1	25	5.9	196	46.0
Low frequency EMF can induce impulses in nerves and muscle cells	LF-EMF	Yes	185	43.3	49	11.5	193	45.2
The specific absorption rate is a measure for the absorption of electromagnetic energy which is transformed to body heat	HF-EMF	Yes	137	32.7	12	2.9	270	64.4
During longer mobile phone calls and unfavourable receiving conditions there might be a temperature increase in the brain of more than one degree	EMF Health HF-EMF	No	126	29.6	161	37.8	139	32.6
The higher the EMF frequency the deeper the penetration into the body	HF-EMF	No	111	26.4	168	39.9	142	33.7

* LF-EMF: Question related to low frequency EMF; HF-EMF: Question related to high frequency EMF; EMF Health: question relating EMF and health risks.

To explore possible latent structures in GPs’ knowledge about EMF, a latent class analysis was conducted. First, the number of expected latent classes was chosen according to the values of the Bayesian information criterion (BIC) and the Akaike information criterion (AIC). The model with minimum value of BIC had two classes; and the model with minimum value of AIC had four classes. As the two criteria identified different models, interpretability of the two models was used to decide on the model with four classes. The GPs were grouped into the four classes using posterior probabilities. The characteristics of the GPs in each of the four classes were described. Multiple linear regression models were used to analyse the effect of latent knowledge classes on GPs’ concern about EMF, as well as the effects of the number of correct answers, the number of incorrect answers and the number of “don’t know”, respectively. The analyses were adjusted for age and age-squared, gender, whether the GP had additional training in alternative medicine (yes/no), and trust in information from the WHO. Statistical analyses were conducted in SAS Version 9.3. For the latent class analysis an additional package (PROC LCA) was used [17].

## 3. Results

The response rate for the long questionnaire was 23.3% (435 participants). The majority of the study population was male, mostly aged between 45 and 55 years, most were classified as specialists in general medicine and 42.1% reported that they had some kind of additional training in alternative medicine. The GP’s knowledge about each of the six EMF items is shown in Table 1. For each item, at least one third of the respondents answered “don’t know” and a considerable proportion gave incorrect answers. The item with the highest percentage of correct answers (58.6%) concerned whether 100 Hertz belongs to the lower frequency range, while the item with the lowest percentage of correct answers (26.4%) concerned the relationship between the frequency of EMF and the capacity to penetrate the body.

**Table 2:** Spearman correlation matrix regarding EMF-knowledge of GPs in Germany in 2008.

	Number of Correct Answers	Number of Incorrect Answers	Number of “Don’t Know” Answers	Self-Estimated Knowledge	Trust in Information from the WHO	Concern about EMF ^†^
**Summary statistics:**						
Mean	2.88	1.22	2.82	3.20	4.17	12.75
Min; max	0; 7	0; 5	0; 7	0; 6	0; 6	6; 24
**Correlation:**						
Number of correct answers	1	0.03	−0.86 *	0.41 *	0.02	−0.08
Number of incorrect answers		1	−0.48 *	0.17 *	0.02	0.15 *
Number of “don’t know” answers			1	−0.46 *	0.00	−0.01
Self-estimated knowledge				1	0.05	−0.01
Trust in information from the WHO					1	−0.16
Concern about EMF						1

^†^ Cronbach’s alpha for the EMF concern score was 0.89; * *p*-value of the Spearman correlation coefficient was below 0.01.

**Table 3:** Results of the latent class analysis, based on probabilities for each response category for the different knowledge questions, GPs in Germany in 2008.

	Correct Knowledge	Don’t Know	LF-EMF *	EMF Health
	0.43	0.13	0.24	0.19
**Response category: correct answer**				
Frequency of 100 Hertz belongs to low-frequency EMF	**0.88**	0.12	**0.68**	0.15
The power from mobile phone is higher the better the EMF reception is.	**0.81**	0.09	0.25	**0.55**
For the German population the mean EMF exposure is far below the legal limits	**0.80**	0.01	0.22	0.48
Low frequency EMF can induce impulses in nerves and muscle cells	**0.57**	0.01	**0.67**	0.13
The specific absorption rate is a measure for the absorption of electromagnetic energy which is transformed to body heat	0.43	0.00	0.27	0.28
During longer mobile phone calls and unfavourable receiving conditions there might be a temperature increase in the brain of more than one degree	**0.53**	0.00	0.04	0.29
The higher the EMF frequency the more the penetration in the body	0.47	0.05	0.20	0.04
**Response category: incorrect answer**				
Frequency of 100 Hertz belongs to low-frequency EMF	0.08	0.02	0.15	0.06
The power from mobile phone is higher the better the EMF reception is.	0.14	0.01	0.28	0.21
For the German population the mean EMF exposure is far below the legal limits	0.06	0.01	0.08	0.07
Low frequency EMF can induce impulses in nerves and muscle cells	0.22	0.00	0.05	0.04
The specific absorption rate is a measure for the absorption of electromagnetic energy which is transformed to body heat	0.06	0.00	0.00	0.02
During longer mobile phone calls and unfavourable receiving conditions there might be a temperature increase in the brain of more than one degree	0.39	0.10	0.44	0.46
The higher the EMF frequency the more the penetration in the body	0.47	0.02	**0.61**	0.26
**Response category: “don’t know”**				
Frequency of 100 Hertz belongs to low-frequency EMF	0.03	**0.86**	0.16	**0.79**
The power from mobile phone is higher the better the EMF reception is.	0.05	**0.90**	0.48	0.23
For the German population the mean EMF exposure is far below the legal limits	0.16	**0.98**	**0.70**	0.45
Low frequency EMF can induce impulses in nerves and muscle cells	0.21	**0.99**	0.27	**0.83**
The specific absorption rate is a measure for the absorption of electromagnetic energy which is transformed to body heat	0.51	**0.99**	**0.63**	**0.70**
During longer mobile phone calls and unfavourable receiving conditions there might be a temperature increase in the brain of more than one degree	0.07	**0.90**	**0.52**	0.25
The higher the EMF frequency the more the penetration in the body	0.06	**0.93**	0.19	**0.70**

* LF-EMF—Low frequency EMF; Bold marked are the values above 0.50.

The correlation matrix in Table 2 shows that the number of “don’t know” answers was significantly negatively correlated with both the number of correct answers and the number of incorrect answers; while the numbers of correct and incorrect answers were uncorrelated. Furthermore as expected, self-estimated knowledge was positively correlated with the number of correct answers and negatively correlated with the number of “don’t know” answers. Self-estimated knowledge was weakly, but significantly, positively correlated with the number of incorrect answers. Trust in risk information provided by the WHO was not correlated with any of the knowledge variables. Besides the number of incorrect answers none of the considered knowledge variables was correlated with EMF concern.

Table 3 shows the results of the latent class analysis. For each knowledge question, the probabilities of each latent class to select each response category are shown. e.g., the probability that a person in the first latent class answers the question. “Frequency of 100 Hertz belongs to low frequency EMF” correctly is 0.88. Thus, Table 3 indicates patterns of response for all four latent classes. Some 43.1% of the GPs gave mainly correct answers; 23.7% of the GPs answered low frequency EMF questions correctly; 19.2% gave answers to the questions relating EMF with health risk but answered the remaining items mainly with “don’t know”; finally, 14.0% nearly always answered “don’t know”. The third group answering only the questions relating EMF with health risk is not clearly defined with regard to their opinion. They answered the questions relating EMF and health risk (3 and 6) correctly and/or incorrectly and all the other questions with “don’t know”; in this way some answered dramatizing regarding EMF as a health risk, while others answered correctly, *i.e.*, not dramatizing. For further presentation, the four groups are labelled: correct knowledge GPs; low frequency EMF GPs, EMF health GPs, don’t know GPs, respectively.

Table 4 presents demographic characteristics as well as knowledge and concern about EMF of the GPs in each of the four groups. Male GPs are overrepresented among the correct knowledge GPs, and the youngest age group is overrepresented among the dramatizing only GPs, while the age group 55–64 years of age is overrepresented in the low frequency EMF group. The number of correct answers varies markedly between the four latent groups but very small differences are seen with regard to self-estimated knowledge, trust in risk communication from the WHO, and concern about EMF.

**Table 4:** Characteristics of GPs in each of the latent classes, GPs in Germany in 2008.

	Correct Knowledge	Don’t Know	LF-EMF *	EMF Health	Total
Overall [*n* (%)]	186 (43.09)	60 (13.05)	102 (23.65)	82 (19.20)	430
Gender [*n* (%)]					
Male	139 (74.73)	30 (50.00)	63 (61.76)	43 (52.44)	275 (63.95)
Female	47 (25.27)	30 (50.00)	39 (38.24)	38 (47.56)	155 (36.05)
Age-groups [*n* (%)]					
<45 years of age	28 (15.22)	13 (21.67)	13 (12.87)	21 (25.61)	75 (17.56)
45–54 years of age	73 (39.67)	18 (30.00)	36 (35.64)	31 (37.80)	158 (37.00)
55–64 years of age	67 (36.41)	22 (36.67)	43 (42.57)	21 (25.61)	153 (35.83)
>64 years of age	16 (8.70)	7 (11.67)	9 (8.91)	9 (10.98)	41 (9.60)
Knowledge [median (5–95 percentile)]					
Number of correct answers	4.0 (3–6)	0.0 (0–1)	2.0 (1–4)	2.0 (0–3)	4.0 (3–6)
Number of wrong answers	1.0 (0–3)	0.0 (0–1)	2.0 (0–3)	1.0 (0–3)	1.0 (0–3)
Self-estimated knowledge	3.5 (2–6))	3.0 (1–5)	3.0 (2–5)	3.0 (1–5)	3.5 (2–6)
Confidence in WHO	4.0 (2–6)	4.0 (1–6)	4.0 (2–6)	4.0 (2–6))	4.0 (2–6)
Concern [median (5–95 percentile)]					
Concern about EMF	12.0 (6–20)	13.0 (6–19)	12.0 (6–20)	13.0 (6–20)	12.0 (6–20)

* LF-EMF—Low frequency EMF.

The associations between GPs’ knowledge and concern about EMF are shown in Table 5. No statistically significant differences in concern about EMF are seen between the correct knowledge class and the other three latent classes. The number of correct answers does not affect the concern about EMF significantly. However, the number of incorrect answers increases the concern about EMF substantially and significantly.

**Table 5:** Multiple linear regression models on the outcome “EMF concern” considering numbers of correct and incorrect answers, and latent knowledge classes, GPs in Germany 2008.

Model		EMF Concern	
		Beta *	95% CI
1	Latent classes:		
	Correct knowledge	Reference	-
	Don’t know	−0.13	−1.39; 1.13
	LF-EMF	0.72	−0.30; 1.74
	Dramatizing only	0.31	−0.81; 1.43
2	Number of correct answers	−0.18	−0.41; 0.04
3	Number of incorrect answers	**0.61**	**0.24**; **0.98**

* adjusted for age, age-squared, sex, trust in EMF information provided by WHO, and education in alternative medicine; Bold marked are significant at significance level 0.05.

## 4. Discussion

Our study confirms the results of most existing literature: there is no association between correct knowledge and concern [18,19,20,21,22,23]. However, our results indicate an association between incorrect knowledge and concern. This has—to our knowledge—not been considered before.

Perceptions of EMF risks in GPs were assessed in Austria [24], Switzerland [25], and Germany [11]. In the Austrian survey 95% of the GPs agreed at least to some degree, that EMF may cause illness and 33% were convinced that EMF cause disease. In a Swiss survey, nearly 61.4% of a sample of 342 GPs believed that there are people with health complaints caused by EMF. In the German survey, using the same questions as in the Swiss survey the analysis revealed that 29% of the GPs believed that there are health relevant effects of EMF. This corresponds to the level of EMF concern in the general population in the majority of member states in EU [1] and in Germany [26].

The latent class analysis describes four classes of knowledge structure among the GPs; most of the GPs, 43.1%, gave mainly correct answers; 23.7% answered low frequency EMF questions correctly; 19.2% stated their view on EMF health items but answered the rest of the items mainly with “don’t know”, and finally only 14.0% answered mostly “don’t know”. Considering the complexity of the questions the proportion of correct answers is quite high. However, considering that 43% of the GPs during the last year had at least one patient naming EMF as a potential risk factor, we recommend increasing EMF related knowledge in GPs. Although, it is unclear why there is no association between correct knowledge and risk perception the result is commonly known among persons who are working in the area of risk communication [27]. People’s risk perceptions evolve along qualitatively different lines and are often quite complex, multi-dimensional, affect-loaded, and deeply rooted in value-systems [27,28,29,30]. Also, rather than seeking “knowledge”, many people fall back on trust (or lack of trust) in authorities who communicate about risk [31,32]. These issues put the potential impact of knowledge into perspective and challenge the “knowledge-gap” theory [33], which suggests that increased information in a society is not equally accessible in different socioeconomic subpopulations. Nevertheless, knowledge is relevant in connection with informed decision-making which to some extent depends on people having the necessary information to make their decisions. Further, knowledge plays an important part in theories on behavioural change incorporating the connection between knowledge and risk perception, e.g., the health belief model [34], social cognitive theory [35] and protection motivation theory [36]. However, the empirical evidence on the impact of knowledge on risk perception suggests mainly that there is no association between knowledge and risk perception [18,19,20,21,22,23,37,38,39,40].

Considering the effect of knowledge in different ways—in our case as incorrect knowledge—revealed an association between the number of incorrect answers and EMF concern. Although it might seem surprising, it does not conflict with the lack of association between correct knowledge and concern about EMF. Particularly in the context of EMF risks there are some possible explanations as to why incorrect knowledge and concern about EMF might be associated. The first explanation for the association might be that until now no conclusive evidence on the health risk of mobile phone technology is available [41], although it has been discussed that, if there is a risk, it is likely to be low [42]. The presentation of scientific evidence can sometimes appear contradictory and polemic, which may give rise to different discussions and different risk opinions. Polemic presentations may even lead to misconceptions of well-known facts. This implies that particularly incorrect answers including the dramatizing answers may be associated with increased EMF concern. Indeed, a sensitivity analysis showed that the number of incorrect answers from the questions relating EMF and health was associated with concern (data not shown). A second explanation may be that the factual understanding of EMF, their physical properties and their potential effect on organisms is complex. Until now, from the physical, physiological, and biological point of view, there is no mechanism available which might explain a health risk of EMF below the scientifically accepted protection limits [42]. Knowledge in persons with misconception about physical, physiological, and biological effects of EMF might easier be capable to explain unhealthy effects of EMF and will therefore be more tightly connected to their EMF health concern.

The key purpose of risk communication is not only knowledge transfer but also dialogue, attention to the nature of the risk (different risk perceptions) and scientific uncertainty. However, risk communication without knowledge transfer is futile, and the message may lose credibility. In risk communication it is recommended to meet the persons at the level they are at, taking their existing knowledge as well as their misconceptions into account [43]. The present result, that incorrect knowledge is associated with EMF health concern, underlines that individual misconception is an important determinant of risk perception and may affect risk communication. In addition, the presented results support the importance of dialogue in risk communication as this allows for identifying and dealing with misconceptions. For GPs it should be recommended that dialogue about EMF health risks becomes a part of their medical professional training.

The results of our analysis are limited by some factors. Firstly, due to the cross sectional design it can only be concluded that there is an association but the direction of the relationship cannot be deduced. That is, it is not known whether incorrect knowledge leads to concern about EMF or a specific concern leads to misconception. Cohort or interventional studies are necessary to clarify this. Secondly, the response rate in our study (23.3%) is low. This is a common problem in surveys with GPs, and the response rate is similar in other surveys [24,25,44]. Therefore, the results may not be representative and might be affected by some selection bias. For example, it has been shown that GPs with additional education in alternative medicine are more often concerned about EMF [12]. However, in the short questionnaire survey of our study with a response rate of 49.1%, we collected information about alternative medicine; there were no differences in prevalence’s of surgeries with alternative medicine between the long and short questionnaire in our survey [3]. Furthermore, we reviewed the Internet homepages of a random sample of GPs from one regional association (*n* = 251, Baden-Württemberg), and we found 41% of them naming additional education in alternative medicine, which is also similar to our overall results. However, there might still be some selection bias affecting our results, and further research is essential to increase participation rates in GPs. Short questionnaires may help to increase the response rate [45], but further developments to reduce barriers of answering questionnaire studies are needed.

An additional limitation is that the knowledge questions were related to technical and general knowledge about EMF and did not concern hypersensitivity diagnosis and treatment. The selection of questions was based on our primary interest in GPs’ general knowledge on EMF. However, further research should focus on GPs’ knowledge relating to diagnostic and treatment of hypersensitivity.

## 5. Conclusions

In conclusion, less than half of the GPs (43%) were able to answer knowledge questions correctly. This may be a problem since 43% of the GPs treated at least one patient during the last year naming EMF as a potential risk factor for his or her disease. Therefore it might be suggested that an EMF related knowledge dialogue should be part of GPs’ medical professional training.

GPs’ latent knowledge class and number of correct answers are not associated with EMF concern. However, our results suggest that the number of incorrect answers is associated with EMF concern. If other studies confirmed these results, misconceptions in populations should be taken into particular consideration in risk communication.
